# Silencing of HLA class I on primary human hepatocytes as a novel strategy for reduction in alloreactivity

**DOI:** 10.1111/jcmm.14484

**Published:** 2019-06-10

**Authors:** Constança Figueiredo, Felix Oldhafer, Eva‐Maria Wittauer, Marco Carvalho‐Oliveira, Ali Akhdar, Oliver Beetz, Chen Chen‐Wacker, Yuliia Yuzefovych, Christine S. Falk, Rainer Blasczyk, Florian W.R. Vondran

**Affiliations:** ^1^ Institute for Transfusion Medicine Hannover Medical School Hannover Germany; ^2^ Excellence Cluster REBIRTH ‐ From Regenerative Biology to Reconstructive Therapy Hannover Germany; ^3^ ReMediES, Department of General, Visceral and Transplant Surgery Hannover Medical School Hannover Germany; ^4^ German Centre for Infection Research (DZIF) Partner Site Hannover‐Braunschweig Hannover Germany; ^5^ Institute of Transplant Immunology Hannover Medical School Hannover Germany

**Keywords:** cell transplantation, hepatocyte, hepatocyte transplantation, HLA silencing, immune response, Immunomodulation, immunosuppression

## Abstract

In contrast to the whole liver, primary hepatocytes are highly immunogenic. Thus, alternative strategies of immunomodulation after hepatocyte transplantation are of special interest. Silencing of HLA class I expression is expected to reduce the strength of allogeneic immune responses and to improve graft survival. In this study, primary human hepatocytes (PHH) were isolated using a two‐step‐collagenase perfusion‐technique and co‐cultured with allogeneic lymphocytes in terms of a mixed lymphocyte hepatocyte culture. Expression of HLA class I on PHH was silenced using lentiviral vectors encoding for β2‐microglobulin‐specific short hairpin RNA (shβ2m) or non‐specific shRNA (shNS) as control. The delivery of shβ2m into PHH caused a decrease by up to 96% in β2m transcript levels and a down‐regulation of HLA class I cell surface expression on PHH by up to 57%. Proliferative T cell alloresponses against HLA‐silenced PHH were significantly lower than those observed form fully HLA‐expressing PHH. In addition, significantly lower secretion of pro‐inflammatory cytokines was observed. Levels of albumin, urea and aspartate‐aminotransferase did not differ in supernatants of cultured PHH. In conclusion, silencing HLA class I expression on PHH might represent a promising approach for immunomodulation in the transplant setting without compromising metabolic function of silenced hepatocytes.

## 1 INTRODUCTION

Hepatocyte transplantation (HTx) is a promising therapeutic approach as treatment for various liver diseases and a potential alternative for orthotopic liver transplantation (OLT) in certain cases. In fact, HTx even has some advantages over OLT, as is it less invasive and expensive than OLT, cryopreserved cells are immediately available when needed, the native liver can potentially regenerate and multiple patients could be benefit from a single organ.^1^ In 1992, the first HTx in human was performed in Japan^2^ and since then, reports have been published that more than 100 patients with liver disease treated by HTx worldwide.^3^ However, despite observation of encouraging clinical improvements in patients transplanted with allogenic hepatocytes, metabolic diseases are only partially cured and in case of acute liver failure, the outcome is very heterogeneous.^3^ In fact, the long‐term success of HTx is still limited by insufficient engraftment and enduring acceptance of cellular allografts. Effective immunosuppression regimens for hepatocyte transplantation have yet to be identified and most centres use protocols known from OLT, despite the fact that hepatocytes are highly immunogenic compared to the liver itself.^4^

Disparities at human leucocyte antigen (HLA) class I and class II loci remain a relevant obstacle in solid organ transplantation. Hepatocytes constitutively express HLA class I antigens and are able of up‐regulating HLA class II under inflammatory conditions supporting the allogeneic immune response.^5^, ^6^, ^7^ Previously, we have demonstrated the feasibility of silencing HLA class I expression in several cell types such as endothelial cells, fibroblasts or CD34^+^ cells. Furthermore, we showed that the down‐regulation of HLA class I antigens prevents cellular and humoral allogeneic immune responses contributing to an overall improved allograft survival in vitro and in vivo. However, residual expression of HLA class I antigens was shown to be essential for preventing NK cell cytotoxic activity.^8^, ^9^, ^10^ Hence, in this study we evaluated the feasibility of reducing the strength of the alloimmune response towards primary human hepatocytes (PHH) by silencing HLA class I surface expression.

## 2 EXPERIMENTAL PROCEDURES

### 2.1 Hepatocyte isolation and culture

Liver tissue was obtained from patients (n = 15) undergoing partial hepatectomy upon written informed consent (approved by the ethic commission of Hannover Medical School, #252‐2008). Hepatocytes were isolated by a modified two‐step collagenase perfusion as previously reported^11^ and cultured using collagen‐pre‐coated 6‐well plates. After 16‐18 hours culture medium was renewed to remove dead cells and ensure formation of a confluent monolayer.

### 2.2 Lentiviral constructs, vector production and transduction of hepatocytes

A lentiviral vector system was used to deliver short hairpin RNA (shRNA) to silence β2‐microglobulin (shβ2m) expression. A vector encoding for a non‐sense sequence was used as a control shRNA (shNS). The vector was produced as previously described.^8^ Briefly, HEK293T cells were cotransfected with psPAX2, pMD2.G and the vector encoding for the shRNA sequence using calcium phosphate (Sigma‐Aldrich, Steinheim, Germany). Lentiviral vector containing supernatants were harvested 48 hours after transfection, filtered and concentrated by ultracentrifugation at 30 000 g (Optima L‐100 XP, Beckmann Coulter, Krefeld, Germany), for 4 hours. The pellets containing the viral vectors were resuspended in Williams's Medium. For hepatocyte transduction, 1.5 × 10^6^ cells were seeded in a 6‐well plate and infected overnight with the vectors in the presence of 8 µg/mL protamine sulphate (Sigma‐Aldrich). Afterwards, hepatocytes were washed and cultured with fresh culture medium.

### 2.3 Analysis of beta2‐microglobulin transcript levels

Total RNA was isolated from hepatocytes (RNeasy Mini Kit, Qiagen, Hilden, Germany) and reverse transcribed to cDNA using the high‐capacity cDNA reverse transcription kit (Applied Biosystems, Darmstadt, Germany). Beta2‐microglobulin (β2m) mRNA levels of hepatocytes were analysed as described before.^12^ Briefly, the native or genetically modified hepatocytes were harvested and total RNA was isolated using the RNeasy Mini Kit (Qiagen, Hilden, Germany) according to the manufacturer's instructions. Reverse transcription of RNA into cDNA was performed with the high‐capacity cDNA reverse transcription kit (Applied Biosystems, Darmstadt, Germany). β2m transcript levels were analysed by real‐time PCR using specific predesigned TaqMan Gene Expression Assays (Hs00984230_m1; Thermo Fisher). Expression values were calculated as ratio to glyceraldehyde‐3‐phosphate dehydrogenase (GAPDH, Hs02758991_g1; Thermo Fisher), which was used as internal control (relative quantification [RQ]). Measurements were performed with PHH from n = 3 different donors.

### 2.4 Mixed lymphocyte hepatocyte culture (MLHC)

On the basis of the in vitro model by Bumgardner et al,^13^ we recently described a modified approach of MLHC.^7^ In brief, hepatocytes cultured as monolayers were used as stimulator cells. Allogeneic peripheral blood mononuclear cells (PBMC) from healthy donors were isolated from whole blood by density gradient centrifugation and used as responder following staining with PKH‐26. MLHC was performed in 6‐well plates with 2 mL supplemented Williams Medium E with daily change of 0.5 mL. PHH were seeded at 1.5 × 10^6 ^cells/well; 5 × 10^6^ naïve responder PBMCs were added on day 0 or cultured alone, as applicable. The following groups were set up: untreated hepatocytes (NC) + PBMC, hepatocytes transduced with a vector encoding for a non‐sense sequence as a control shRNA (shNS) + PBMC and PHH transduced with the vector encoding for the shRNA to silence β2‐microglobulin expression (shβ2m) + PBMC; control groups with hepatocytes or PBMC alone. Culture supernatants were stored at −80°C for cytokine analysis in batch. Regarding the design of these experiments, PHH from a single donor (total n = 5) were used to set up the MLHC with PBMC of one to two different donors (total n = 7), eventually resulting in n = 7 different MLHC experiments.

### 2.5 Flow‐cytometry for analysis of proliferative alloresponses in MLHC

PKH‐26 stained PBMC were analysed on day 10 by flow‐cytometry. Additional staining for CD4 and CD8 was performed to distinguish T cell subpopulations as previously reported.^7^ Furthermore, staining for CD3 and CD56 was performed in order to identify Natural killer (NK) cells. Flow‐cytometric measurements were performed with a FACSCalibur (BD Biosciences) and results were analysed by FACSDiva software.

### 2.6 Cytokine multiplex analyses

Cytokine secretion analyses were performed in cell culture supernatants using a Th1/Th2/Th17 Milliplex kit (Merck Millipore, Schwalbach, Germany) and Luminex 100/200 technology according to the manufacturer's instructions. Cytokine concentrations were calculated using the Xponent software version 3.1 (Thermo Fischer Scientific).

### 2.7 NK cell isolation

NK cells were isolated from PBMC of healthy donors (n = 3) by negative selection using MACS (Magnetic Activated Cell Sorting) technology (Miltenyi‐Biotec) as previously reported.^14^ NK cell purity was determined by FACS as CD3^‐^CD56^+^ lymphocytes and reached >80%. NK cell subsets were analysed using mAb specific for CD56, CD16, CD6 and DNAM‐1 (CD226) as described by Hoffmann et al.^14^

### 2.8 Co‐culture of PHH with NK cells

PHH were incubated with freshly isolated NK cells (6 × 10^5^ cells) at a 1:1 ratio in 24‐well plates in RPMI1640 medium with 10% FBS, Pen/Strep, L‐Glutamine, Na‐Pyruvate either without additional stimulus or with 500 U/mL IL‐2, 100 ng/mL IFN‐α, or a combination of IL‐12 (100 ng/mL) and IL‐15 (100 ng/mL). After 48 hours of co‐culture, NK cells were harvested and stained with a live/dead fluorochrome (yellow dead stain BV570) to exclude dead cells from subsequent phenotypic analyses (shown in Figures 6 and 7). Subset composition and receptor expression was analysed by FACS as described above. Experiments were performed with PHH from n = 3 different donors.

### 2.9 Flow‐cytometric analysis of T and NK cell ligands on PHH

Forty‐eight hours after transduction, hepatocytes were collected and stained with Alexa‐Fluor 647 conjugated anti‐HLA class I antibody (W6/32, murine IgG2a) or isotype control mAb. The cells were acquired by a FACSCanto II and analysed using the FACSDiva (BD Biosciences) and the FlowJo software. Expression of HLA‐E (clone 2D4, rat IgG2, hybridoma), HLA‐DR (L243, murine IgG2a, hybridoma), the adhesion molecule ICAM‐1 (gp89 murine IgG2a hybridoma) and the NK receptor ligands CD155 (PVR IgG2a, Coulter USA) and CD166 (ALCAM) were determined using goat antimouse PE‐labelled secondary antibody and isotype control mAb. Measurements were performed with PHH from n = 4 different donors.

### 2.10 Albumin synthesis

The synthesis of albumin by hepatocytes was assessed using the Human Albumin ELISA Quantitation Set (Bethyl Laboratories) according to the manufacturer's instructions as previously reported.^11^

### 2.11 Aspartate‐aminotransferase activity and urea production

The activity of the aspartate‐aminotransferase (AST) served as a measure for the degree of cell damage, whereas the production of urea served as an indicator for ammonia detoxification. Both parameters were determined in the supernatants of hepatocyte cultures by standardized procedures (Roche Molecular Diagnostics) performed by the central laboratory of Hannover Medical School as previously reported.^11^

### 2.12 In vitro morphology

The morphology of with/without HLA class I silenced PHH or was assessed daily throughout the entire culture period using phase‐contrast microscopy.

### 2.13 Statistical analysis

Statistical analysis was performed with GraphPad Prism 5 (Hersteller). Mann‐Whitney *U* test and paired *t* test were applied as appropriate. Differences were regarded statistically significant with *P* < 0.05. Results were expressed as mean ± SEM unless indicated otherwise.

## 3 RESULTS

### 3.1 Early surface expression of T and NK Receptor Ligands on PHH

**Figure 1:**
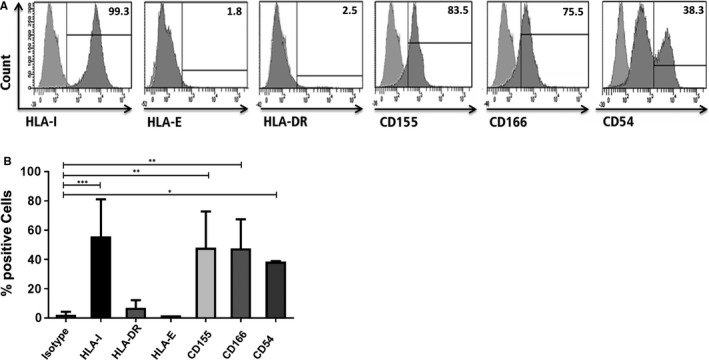
Statistical overview of surface marker expression of PHH. PHH were stained for indicated ligands and analysed by flow‐cytometry. A, Representative dot plots depicting ligand expression, B, bar charts summarizing the results. Data are presented as mean ± standard deviation. (n = 4, *P < 0.05, **P < 0.01, ***P < 0.001)

In order to analyse the impact of HLA class I silencing in this setting, first, the surface expression of HLA class I, the non‐classical class I HLA molecule HLA‐E, HLA‐DR and other ligands like CD155, ALCAM (CD166) and ICAM‐1 (CD54) were analysed on untreated PHH by flow‐cytometry (Figure 1A,B). All molecules contribute to interactions with NK cells and/or T cells and may, thus, influence the interaction of liver epithelial cells with these effector cells. PHH were positive for classical HLA class I (99.3%) but negative for the non‐classical HLA‐E molecule and HLA‐DR They showed weak expression of the DNAM‐1 ligand CD155 and the CD6 ligand CD166. In contrast to these molecules, two populations were observed for ICAM‐1 expression indicating different densities of this adhesion molecule on PHH.

### 3.2 Silencing HLA class I expression on hepatocytes

**Figure 2:**
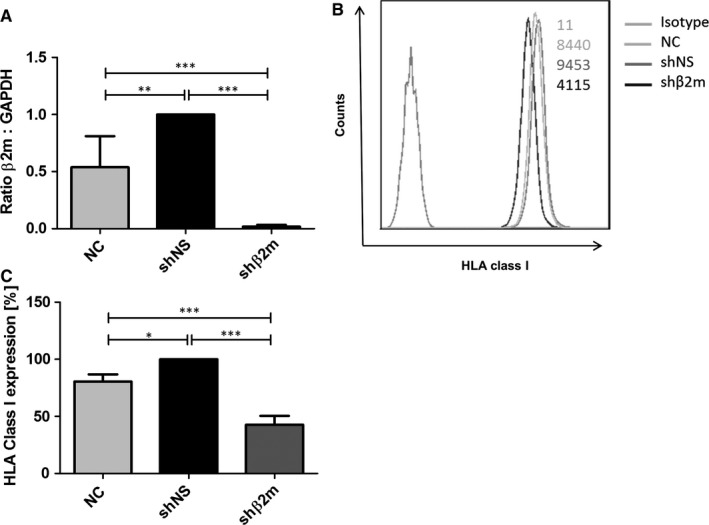
Silencing HLA class I expression on hepatocytes. A lentiviral vector encoding for shβ2m was used to silence HLA class I expression on hepatocytes. Non‐transduced cells or transduced with a vector encoding for a non‐specific shRNA sequence were used as controls. A, Levels of β2‐microglobulin transcripts were detected by real‐time PCR 48h after transduction (n = 5). B, Representative experiment of HLA class I protein levels on genetically modified hepatocytes and controls. Mean fluorescence intensities (MFI) are indicated in the plot for each condition. C, The bar graph depicts mean ± SEM of the level of HLA class I expression on hepatocytes. HLA expression was normalized to the levels detected on hepatocytes treated with the control vector encoding for the non‐specific shRNA (shNS). (n = 3, *P < 0.05, **P < 0.01, ***P < 0.001)

The transduction of hepatocytes with the lentiviral vector encoding for shβ2m caused significant down‐regulation of β2‐microglobulin transcript levels of 96% (*P* < 0.001) in comparison to shNS (Figure 2A). This contributed to a reduction in HLA class I cell surface protein levels of 57% (*P* < 0.001) in the hepatocytes expressing shβ2m in comparison to shNS (Figure 2B,C).

### 3.3 Albumin synthesis, Aspartate‐aminotransferase activity and urea production were not affected by HLA class I silencing

**Figure 3:**
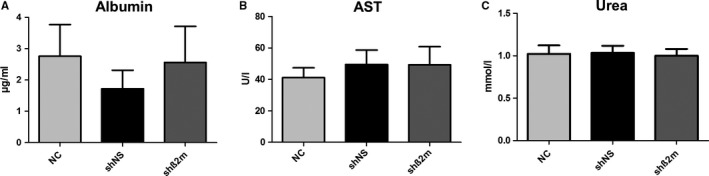
Evaluation of the effect of HLA class I silencing on the functional capacity of PHH in vitro. Bar charts depicting the mean amount of albumin synthesis (A), AST‐leakage (B) and urea production (C) determined on day 10 of hepatocyte cultures in the absence of HLA class I silencing (NC) or after the use of lentiviral vectors encoding for non‐specific shRNA (shNS) and β2‐microglobulin (shβ2m) respectively. Data are presented as mean ± SEM (n  =  7)

To evaluate the potential impact of HLA class I silencing on the functional capacity of PHH, subsequent studies on cultured hepatocytes were performed. As a measure of cell damage, the leakage of AST into the culture supernatants was assayed, but did not show significant differences between the control and the various experimental groups. Regarding the synthesis of albumin (hepatocyte specific function) and the production of urea, there were likewise no significant differences concerning the different experimental groups as summarized in Figure 3. Furthermore, in line with the functional findings, no morphological differences in cultured cells could be observed. Cells of all groups showed the typical morphological appearance of PHH using phase‐contrast microscopy. These were highly prismatic, presented a typical polygonal shape and were either mono‐ or polynucleated (data not shown).

### 3.4 Suppression of proliferative alloresponses in MLHC by silencing of HLA class I expression on hepatocytes

**Figure 4:**
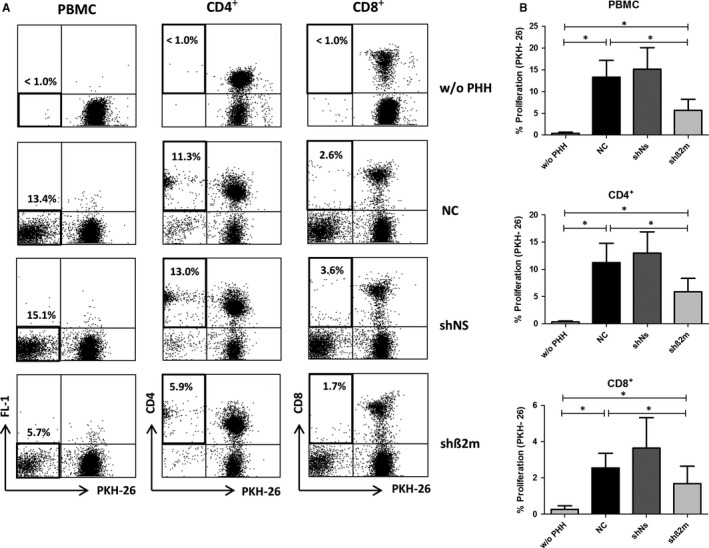
Flow‐cytometric characterization of hepatocyte‐induced T cell alloresponses in MLHC. A, Representative dot‐plots depicting proliferative alloresponses on day 10 of MLHC for PBMC or staining for CD4^+^ and CD8^+^ T cells, respectively, in co‐culture with PHH with or without silencing of HLA class I (NC vs shß2m respectively), also including a control group with a vector encoding for non‐specific shRNA (shNS). Percentages in the left top corner represent the value within the black box. B, Bar charts summarizing the mean percentage of proliferation after 10 d of MLHC. Data are presented as mean ± SEM (n = 7, *P < 0.05)

The novel co‐culture system MLHC was used to characterize the immune responses against allogeneic PHH and the immunosuppressive potential of silencing HLA class I expression on hepatocytes. As described previously,^7^ the immune response induced by allogeneic PHH in vitro was predominantly CD4^+^ T cell driven, whereas only limited proliferation was observed for CD8^+^ T cells (Figure 4A,B). The proliferative alloresponse of PKH‐26 stained PBMC was significantly reduced by silencing HLA class I expression on hepatocytes (5.7% ± 2.5% (shβ2m) vs 13.4% ± 3.8% (NC); *P* = 0.0156; Figure 4A,B). In detail, CD4^+^ and CD8^+^ T cells both showed significant reduction in alloproliferation towards silenced hepatocytes (shβ2m), when compared with the untreated control group (NC) (5.9% ± 2.5% vs 11.3% ± 3.5%, *P* = 0.0156 and 1.7% ± 1.0% vs 2.6% ± 0.8%, *P* = 0.0333 respectively). At the same time the frequency of CD3^‐^CD56^+^ NK cells was low in general and not significantly altered upon silencing HLA class I expression in hepatocytes (1.2% ± 0.8% vs 1.3% ± 0.4% for shβ2m vs NC, *P* = 0.8842, n:3).

**Figure 5:**
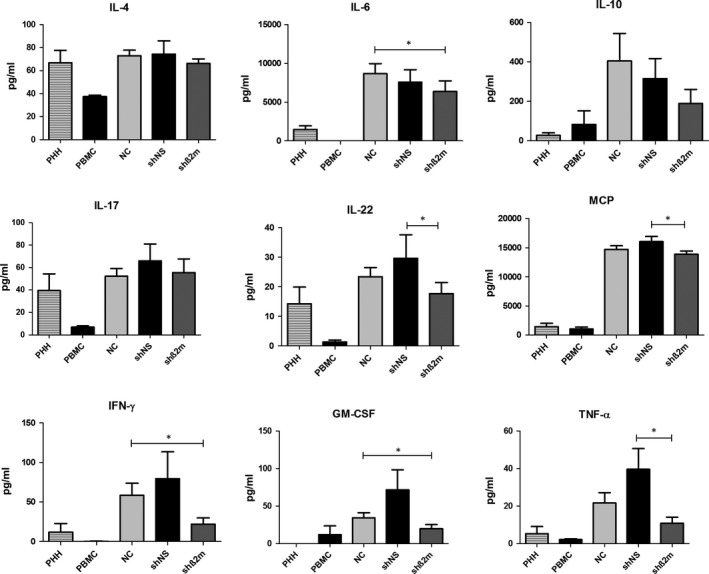
Determination of hepatocyte‐induced cytokine responses and characterization of the suppressive effects of HLA class I silencing. Bar charts summarizing the results of cytokine analyses determined in culture supernatants on day 10 of MLHC. Expression levels of selected cytokines for respective experimental groups without co‐culture of PHH (PHH and PBMC alone) as well as in the presence of PHH (non‐silenced PHH (NC) or after the use of lentiviral vectors encoding for non‐specific shRNA (shNS) and β2‐microglobulin (shβ2m) respectively) are represented as mean ± SEM (n = 4, *P < 0.05)

Measurement of cytokine levels on day 10 in supernatants of MLHC was performed to further characterize the immune reaction of PBMC on PHH with and without HLA class I silencing (Figure 5). Cytokine levels were highest in the non‐transduced (NC) or control shRNA (shNS) groups. HLA class I silencing significantly suppressed pro‐inflammatory cytokines such as IL‐6 (*P* = 0.0244), Interferon‐gamma (IFN‐γ) (*P* = 0.0318) and Granulocyte‐macrophage colony‐stimulating factor (GM‐CSF) (*P* = 0.0075) compared to MLHC with non‐HLA class I silenced PHH (NC). T‐helper (Th) 2‐associated cytokine IL‐10 was also suppressed; however, the observed differences did not reach statistical significance.

### 3.5 NK cells are already suppressed by wild‐type PHH after 48 hours of culture

**Figure 6:**
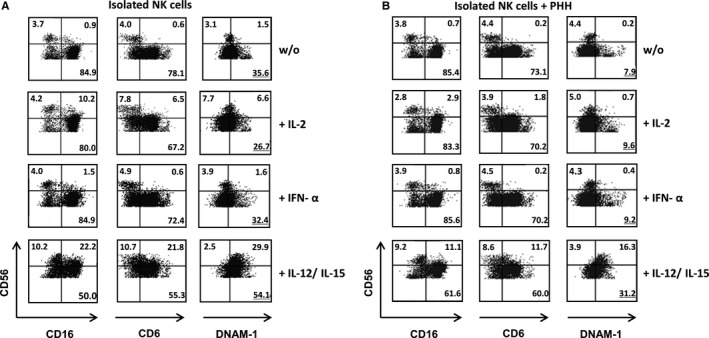
Analysis of the effect of co‐culture of PHH with NK cells. Influence of PHH on the CD16, CD6 and DNAM‐1 expression of NK cells. 6 × 10^5^ MACS isolated NK cells were co‐cultured or cultured alone for 48 h with 6 × 10^5^ PHH, either without any additional stimulus, with 500 U/mL IL‐2, 100 ng/mL IFN‐α or a combination of IL‐12 (100 ng/mL) and IL‐15 (100 ng/mL) at 37°C. NK cells were carefully harvested and analysed by flow‐cytometry for CD56, CD16, CD6 and DNAM‐1. One of three representative experiments is shown without (A) or with PHH (B) co‐culture

**Figure 7:**
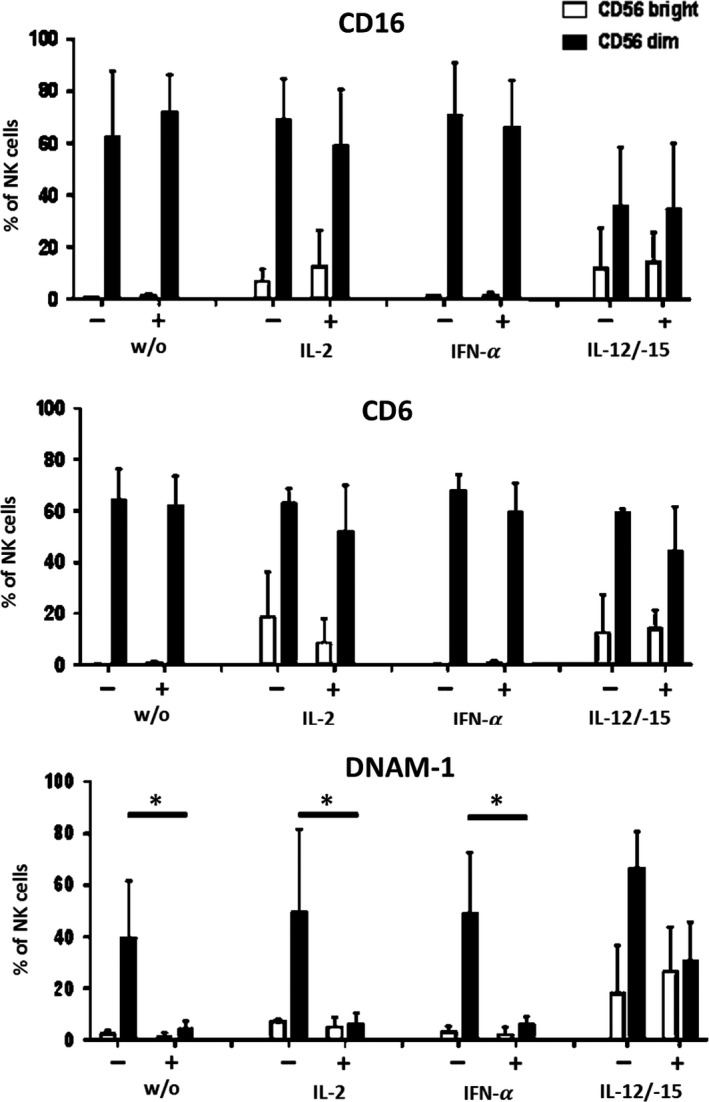
Analysis of the effect of co‐culture of PHH with NK cells. Influence of PHH on the CD16, CD6 and DNAM‐1 expression of NK cells. Bar charts summarizing the results with respect to surface expression of these receptors upon co‐culture with PHH. Black bars represent CD56^dim^ NK cells and white bars represent CD56^bright^ NK cells. Black minus: NK cells without co‐culture of PHH; Black plus: NK cells with co‐culture of PHH. Data are presented as mean ± standard deviation (n = 3, *P < 0.05)

Silencing of HLA class I bears the potential of inducing an NK‐cell‐driven immune response.^9^ Since NK cells in our experimental setting were only observed at very low numbers on day 10 of MLHC, we further analysed the direct influence of PHH on NK cells in the early phase of co‐culture. Specifically, the effect of PHH on the surface expression of NK receptors like CD16, CD6 and DNAM‐1 was investigated in the presence or absence of cytokine stimulation. Under non‐stimulated conditions, CD16 and CD6 expression on CD56^dim^ and CD56^bright^ NK cells was unaltered in the presence of PHH (Figure 6) compared to NK cells alone. In contrast, DNAM‐1 expression on CD56^dim^ NK cells was reduced substantially (without PHH 35.6%, with PHH 7.9%). This effect was also seen for stimulation with IL‐2 or IFN‐α. In contrast, the presence of IL‐12/IL‐15 protected NK cells from DNAM‐1 down‐regulation and instead, resulted in enhanced DNAM‐1 levels on CD56^dim^ and CD56^bright^ NK cells (Figure 7). These results indicate that contact to PHH affects NK cell receptor expression at an early point of co‐culture, which as early as and, may ultimately result in the low number of NK cells present after 10 days of co‐culturing PBMC and PHH. Addition of exogenous pro‐inflammatory cytokines, that is IL‐12 and IL‐15 can prevent receptor modulation and eventually support NK cell survival. For all culture conditions, the % of excluded dead cells was below 10%. Mild, less than twofold, expansion of NK cells was observed in the presence of IL‐2, IFN‐a and IL‐12/15 without statistically significant differences.

## 4 DISCUSSION

In this study, we present a novel and efficient gene regulatory strategy to decrease immunogenicity of allografts in hepatocyte transplantation. We investigated, to our knowledge for the first time, the strength of the allogeneic immune response of stably HLA I‐silenced human hepatocytes in vitro. Current immunosuppressive medications target the immune system of the recipient in a general manner, which may cause substantial systemic effects. Therapies targeting the donated organ prior to transplantation remain unexplored and might be safe and highly desirable alternative approaches.^15^ Modulation of the graft to minimize rejection may allow reduction in the level of systemic immunosuppression usually required to prevent graft rejection. Thus, rejection of the highly immunogenic hepatocytes might be prevented without the risk of severe side effects of high doses of systemic immunosuppression such as nephrotoxicity, hypertension, diabetes and increased risk for opportunistic infections and malignancy.^16^ Freshly isolated PHH are known as HLA class I^+^, class II^−^, which was confirmed by our experiments, and can thereby, stimulate allospecific T cells upon co‐culture. We have shown previously, that allogeneic PHH induce a T cell‐mediated immune reaction of PBMC in vitro, surprisingly as a proliferative response of primarily CD4^+^ T cells. However, CD8^+^ T cells showed significant up‐regulation of the early activation marker CD69.^7^ Further in vitro studies from Gao et al, using human hepatocytes, showed that CD40/CD40 ligand interactions induce T cell activation and apoptosis, providing further evidence that hepatocytes can promote antigen‐presenting cell function.^17^ Allen et al also described the role of the T cell response to a mismatched HLA class I antigen in a patient with Crigler‐Najjar type 1.^18^ Hence, despite the small number of treated patients, there is an increasing evidence suggesting that disparities at HLA class I and class II loci might compromise the overall outcome after HTx. Previously, we have demonstrated that HLA class I‐silenced cells have a superior capacity to escape antibody‐mediated, complement‐dependent and cellular‐dependent cytotoxicity in vitro and in vivo.^9^, ^10^, ^19^ Moreover, recent studies have shown that CD8^+^ T cells play an important role in cellular alloimmune responses against the transplanted hepatocytes.^20^, ^21^ The decrease in proliferation observed in our MHLC, when HLA class I expression was down‐regulated, may indicate a lower activation of both CD4^+^ and CD8^+^ T cells. For silencing of HLA class I, down‐regulation of CD8^+^ T cell‐driven immune responses would be expected and indeed we could observe significant down‐regulation of CD8^+^ T cell proliferation despite the fact that in our MLHC the CD4^+^ T cell subpopulation is the main contributor of alloproliferation. In a recent publication, we showed that regulatory T cells were not capable to reduce proliferation of CD8^+^ cells in contrast to a suppressive effect on CD4^+ ^T cells in this setting,^7^ emphasizing the potential advantage of HLA silencing as a different approach towards immunomodulation, when compared with a cell‐based therapeutical approach with regulatory T cells.

Furthermore, cytokines are important mediators and amplifiers of alloimmune responses after transplantation.^22^, ^23^ In fact, after hepatocyte transplantation specific cytokine storms have shown to regulate tolerogenic responses or rejection.^24^ Remarkably, a significant decrease in the secretion of pro‐inflammatory cytokine IL‐6 was detected when HLA‐silenced hepatocytes were used as targets. This suggests that HLA class I‐silenced hepatocytes induce a weaker alloimmune response in comparison to fully HLA‐expressing cells and might explain the reduced proliferation of CD4^+^ T cells previously shown to be associated with HLA class II up‐regulation on hepatocytes.^5^, ^7^ The tolerogenic cytokine IL‐10 was also found to be increased during strong rejection events as a negative feedback mechanism of the immune response.^25^, ^26^, ^27^ Levels of IL‐10 were decreased in MHLC using HLA‐silenced hepatocytes, which could be related to the lower activation state of immune cells as demonstrated by the lower proliferation activity and pro‐inflammatory cytokine secretion profiles. Hence, our data support the assumption that silencing HLA class I expression may ameliorate the alloimmune response towards hepatocytes.

NK cell‐mediated cell lysis in the absence of HLA I molecules is a severe risk associated with HLA class I silencing.^9^ In our study, we could only see marginal numbers of NK cells surviving long‐term (10 days) co‐culture of PBMC and PHH. When looking at NK cells cultured alone with PHH in the presence of cytokines (IL‐2, IFN‐α or IL‐12/IL‐15) we could observe that contact to PHH leads to an early down‐regulation of the activating NK cell receptor DNAM‐1 and further suppresses secretion of IFN‐γ and TNF‐α, which might explain the small numbers of NK cells and their inferior role in our MLHC experiments. Furthermore, in previous studies, we demonstrated that the residual expression of HLA class I is sufficient to prevent NK cell cytotoxicity.^9^, ^10^

In order to allow a permanent suppression of HLA required for a stable decrease in the hepatocytes` immunogenicity, a lentiviral vector was used for the delivery of the HLA‐specific shRNA. The use of lentiviral vectors is intrinsically associated with safety concerns associated with possible insertional mutagenesis or genotoxic adverse events.^28^ However, several progresses have been made to reduce safety risks of gene therapeutic approaches such as the use of self‐inactivating vectors, suicide genes, integration in safe harbours, enhancer‐blocking insulators, or microRNA targeting of vectors resulting in an increasing number of phase I/II clinical trials.^28^, ^29^ HLA‐silenced hepatocytes morphology and functionality remained comparable to non‐modified hepatocytes or just expressing a non‐specific shRNA. This suggests that silencing HLA expression or the transduction process does not affect the quality of the hepatocytes. Furthermore, the use of inducible promoters may give the opportunity in the future to re‐express HLA if desired in case of infection or cancer.^30^

Main limitation of this study is the in vitro nature of the experiments and the restriction on hepatocyte transplantation, whereas OLT still represents the gold standard therapy for chronic and acute liver failure as well as liver‐based metabolic disorders. Especially since liver transplantation is performed in patients with hepatocellular carcinoma, and there are encouraging reports and trials about OLT as a treatment option for colorectal liver metastases and hilar cholangiocarcinoma in selected cases,^31^, ^32^ immunosuppression sparing strategies are of special interest to reduce the probability of tumour recurrence, also in OLT. Lastly, the study is limited to HLA class I silencing, therefore silencing of HLA class II or both class I and II might further reduce the immunogenicity of hepatocytes, therefore further studies on this issue are necessary.

In conclusion, silencing of HLA class I on PHH might represent a promising approach for immunomodulation in the transplant setting without compromising metabolic function of the silenced hepatocytes. However, further studies are necessary to analyse the relevance of this approach in vivo.

## CONFLICT OF INTEREST

The authors confirm that there are no conflicts of interest.

## Data Availability

The data that support the findings of this study are available from the corresponding author upon reasonable request.
